# Supplementary material for: An Easy-to-Fabricate Cell Stretcher Reveals Density-Dependent Mechanical Regulation of Collective Cell Movements in Epithelia
Source: Cell Mol Bioeng. 2021 Jul 28;14(6):569–81. doi: 10.1007/s12195-021-00689-6 (PMC8630312; doi:10.1007/s12195-021-00689-6)
Supplement: Supplementary file 3 — Supplementary file3 (DOCX 3411 kb) [file 12195_2021_689_MOESM3_ESM.docx]

**Supplemental Figures:**

**
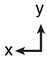

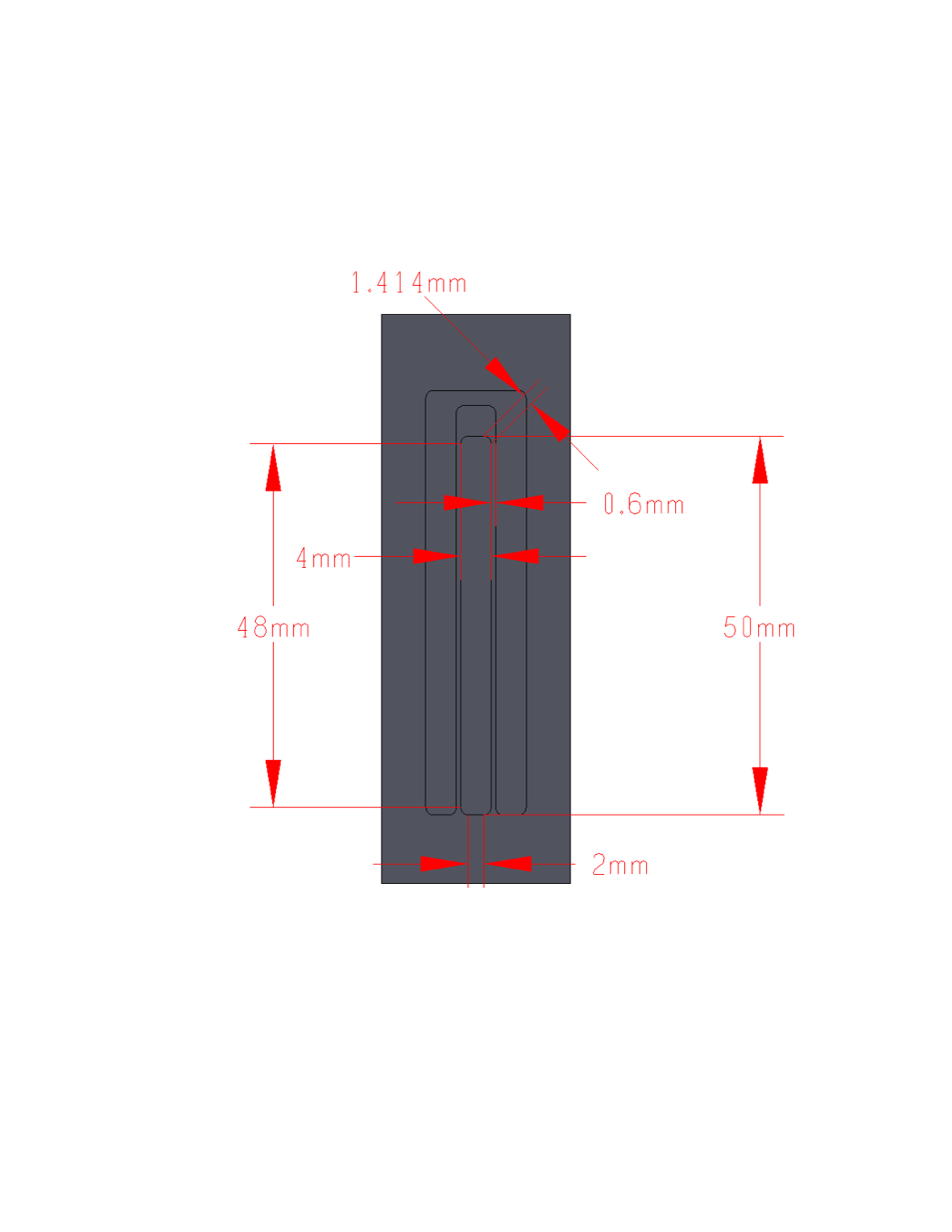
**

**Supplemental Figure 1:**

Dimensions of the 3-D printed mold of the cell stretching device looking down on the mold in a x-y orientation. The cell culture chamber is in the center and is 4 mm x 48 mm and it is surrounded by the vacuum chamber that is 4 mm wide. In between the vacuum chamber and cell culture chamber is a 0.6mm wide sidewall that separates the two chambers.

A. B.
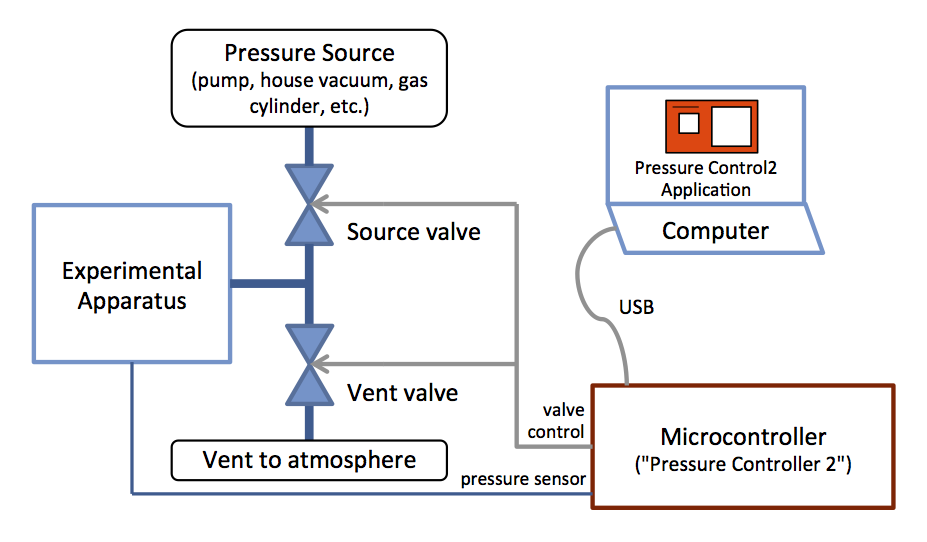


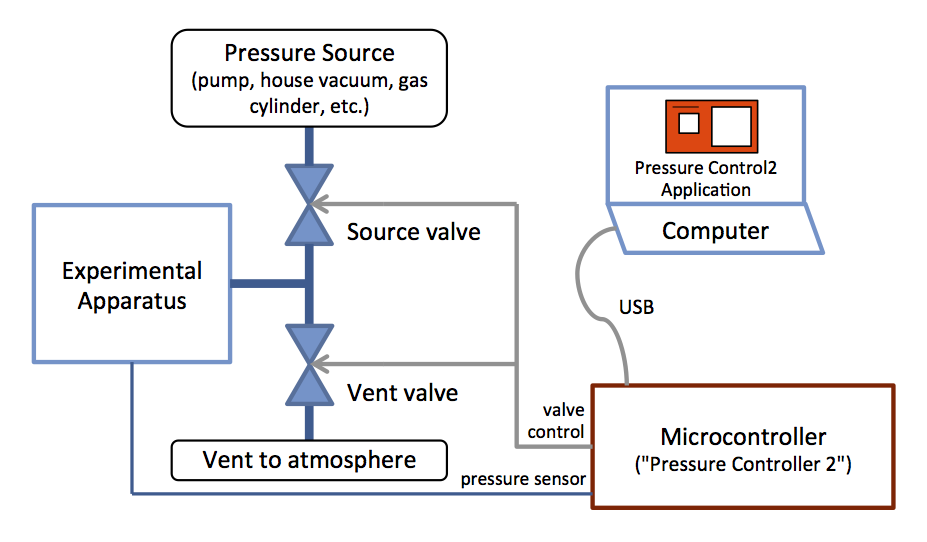


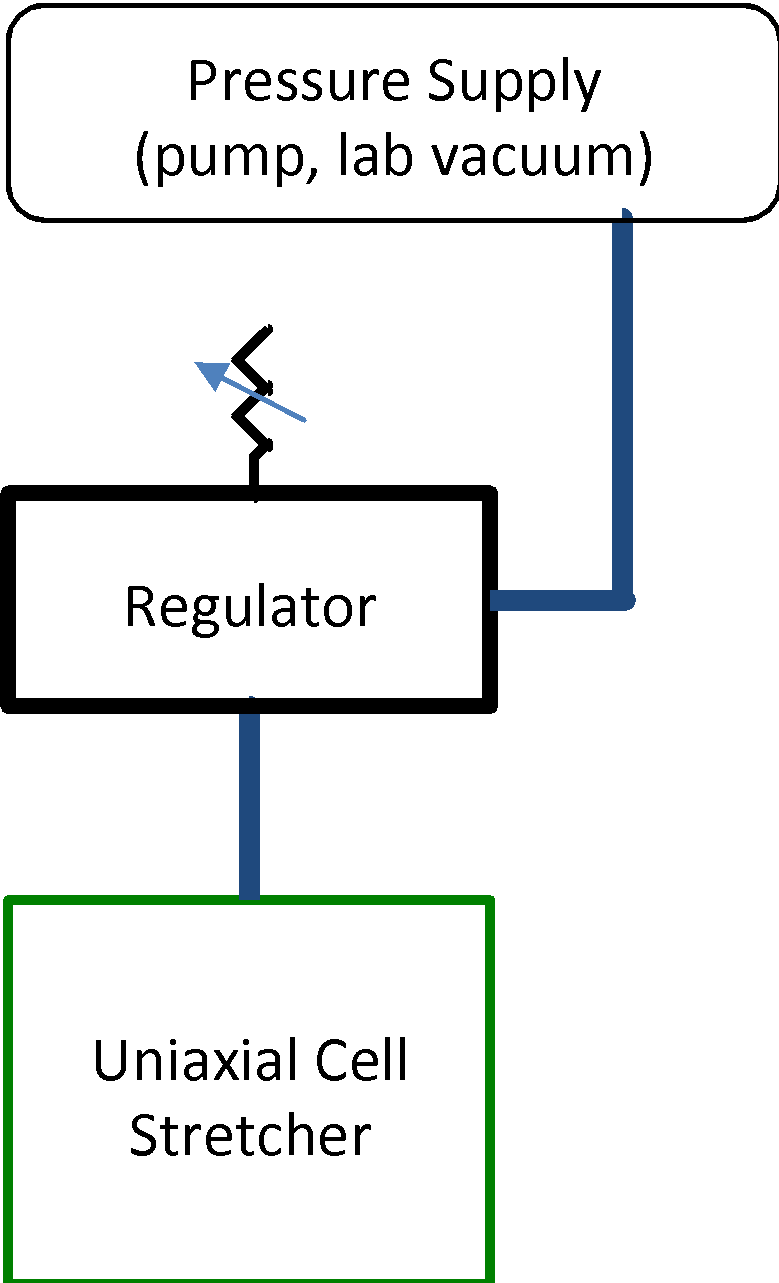


**Supplemental Figure 2:**

Schematic diagram of the two types of pressure regulators used in the experiments.

A. Schematic diagram of the mechanical regulator, High Precision Vacuum Regulator-P3RA171, from Parker. The vacuum regulator connects the in-house vacuum line, with a vacuum pressure of 90 kPa, to the device.

B. Schematic diagram of the electronic pressure controller from Red Dog Research. This electronic controller connects the in-house vacuum line to a valve controller that is connected to a laptop. The application on the computer can control the valves to within 0.25 kPa of the setpoint and it has the capacity to execute pre-programmed pressure waveforms, and hence modulate strain in a variety of ways, including sinusoid waveforms. The frequency response of the system depends on vacuum pressure available. For our measurements, with maximum vacuum pressure of 90 kPa, the system was able to create a sinusoidal pressure profile with a frequency of 1 Hz and amplitude of 15 kPa, which corresponds to a strain of about 7%. Faster profiles are possible at lower amplitudes.

**
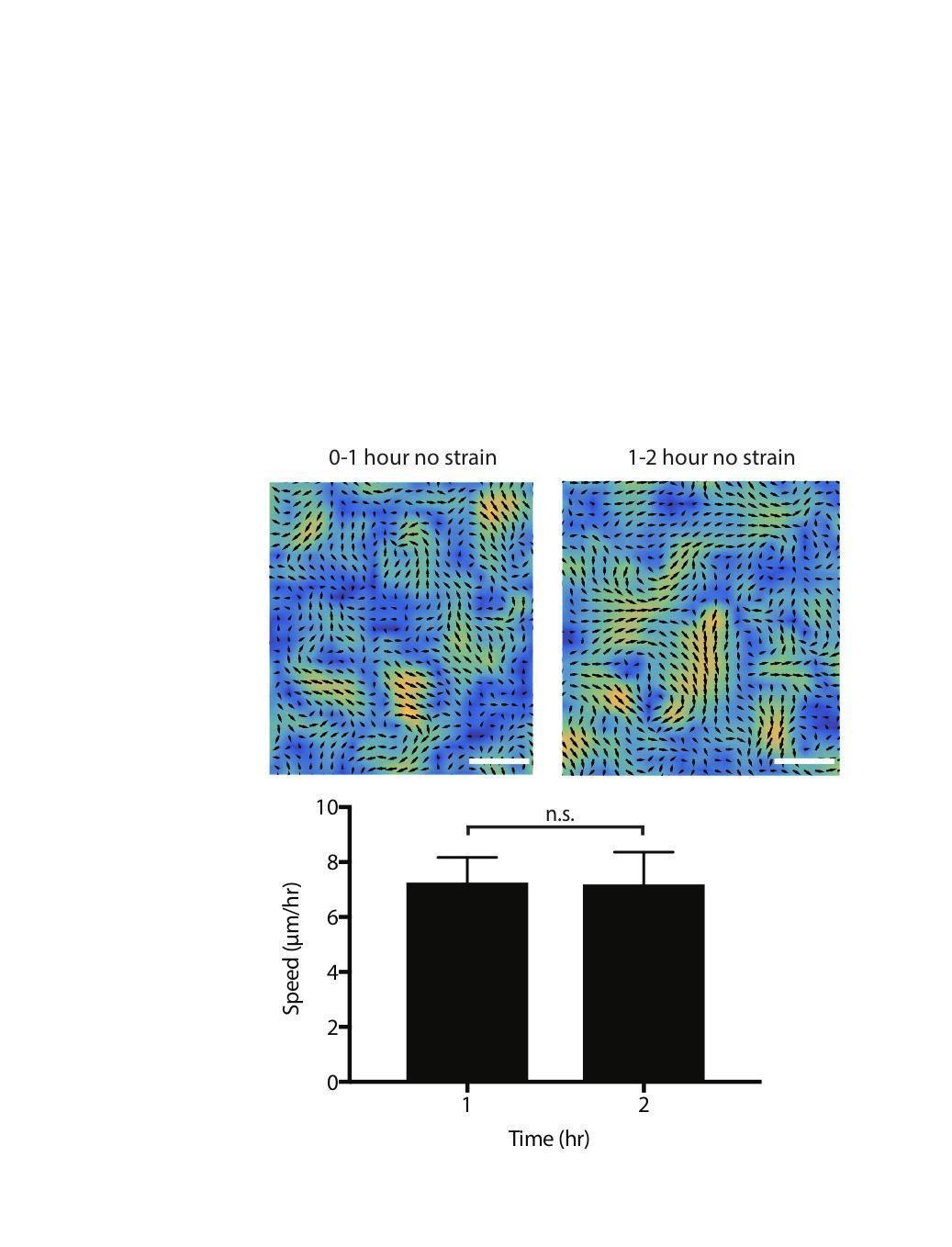
**


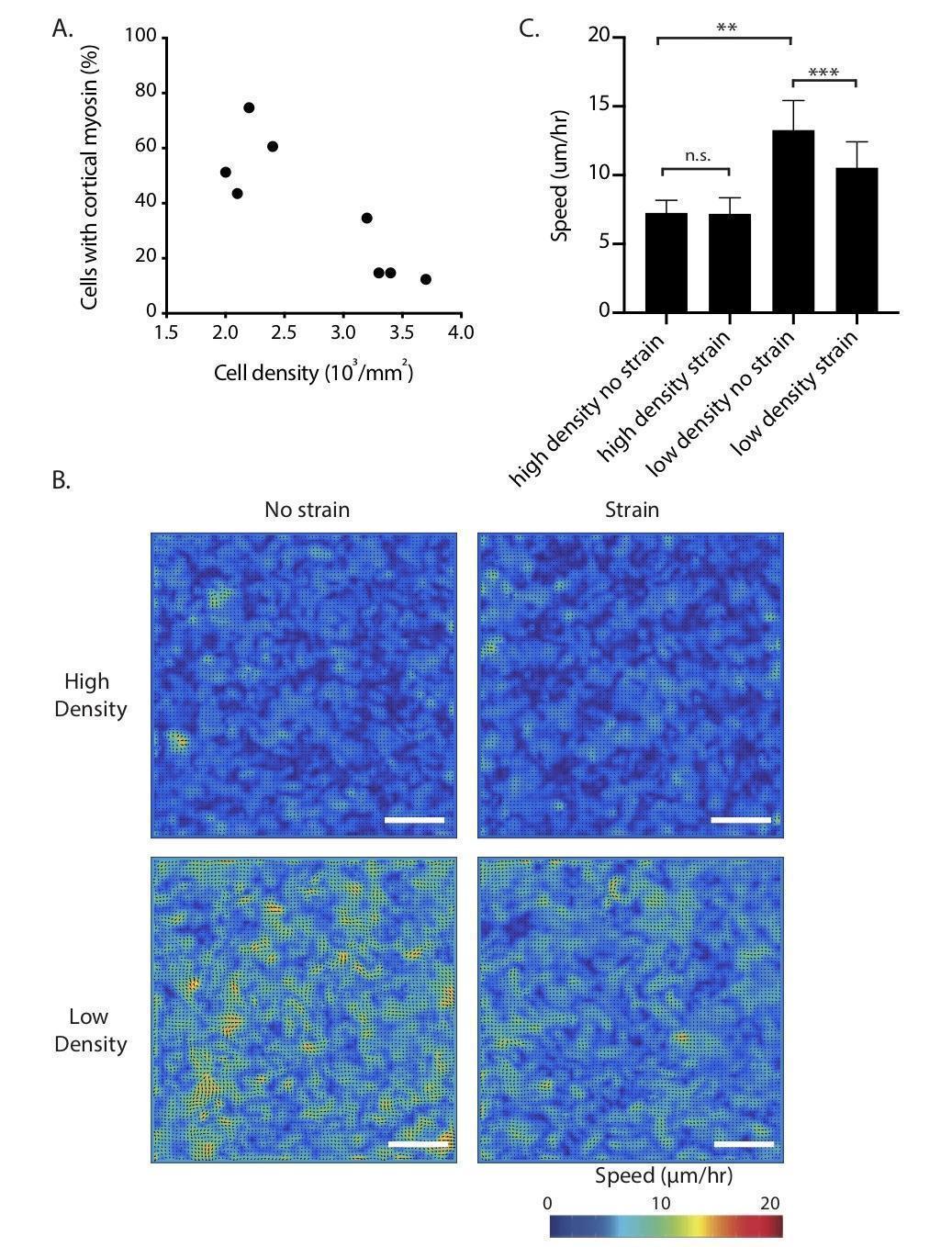


**Supplemental Figure 3:**

Mean velocities of MDCK cell monolayers under no strain conditions over the course of 2 hr. Cell monolayers were imaged with phase contrast every 10 min at 5x magnification. The cell monolayer was imaged for 1 hr followed by another hour with no strain. The collective migration velocities were measured with PIV (particle image velocimetry). Scale bars: 50 μm.

**
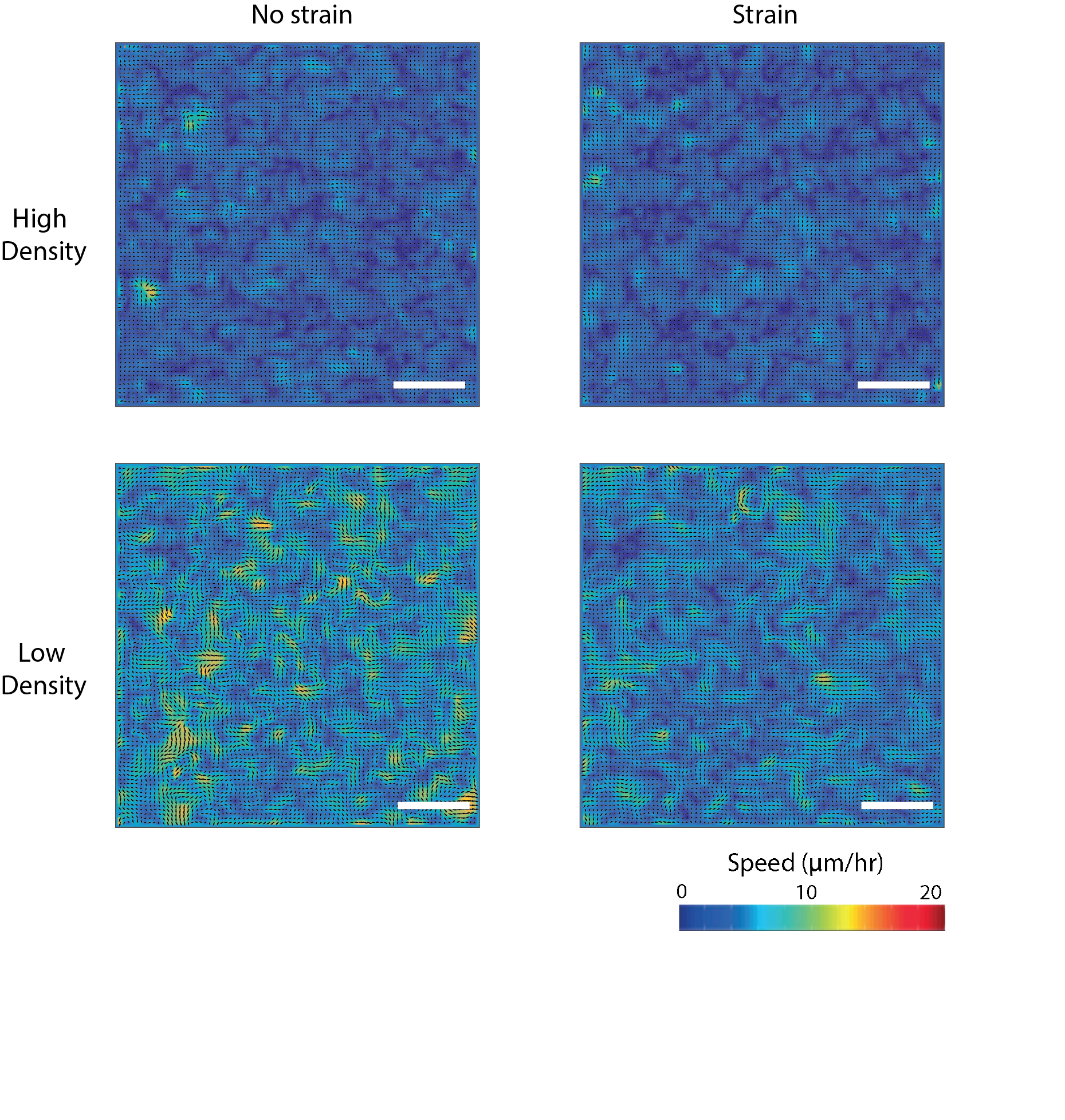
**

**Supplemental Figure 4:**

Representative mean velocity maps from 1 hr of imaging, calculated from the phase contrast images taken every 10 min at 5x magnification of both low-density (2.0 x10^3^/mm^2^ –2.5 x10^3^/mm^2^) and high-density (3.0 x10^3^/mm^2^ –4.0 x10^3^/mm^2^) monolayers. The cell monolayers were imaged for 1 hr followed by another hour with or without strain. The collective migration velocities were measured with PIV (particle image velocimetry). Scale bars: 150 μm.

A.

B.
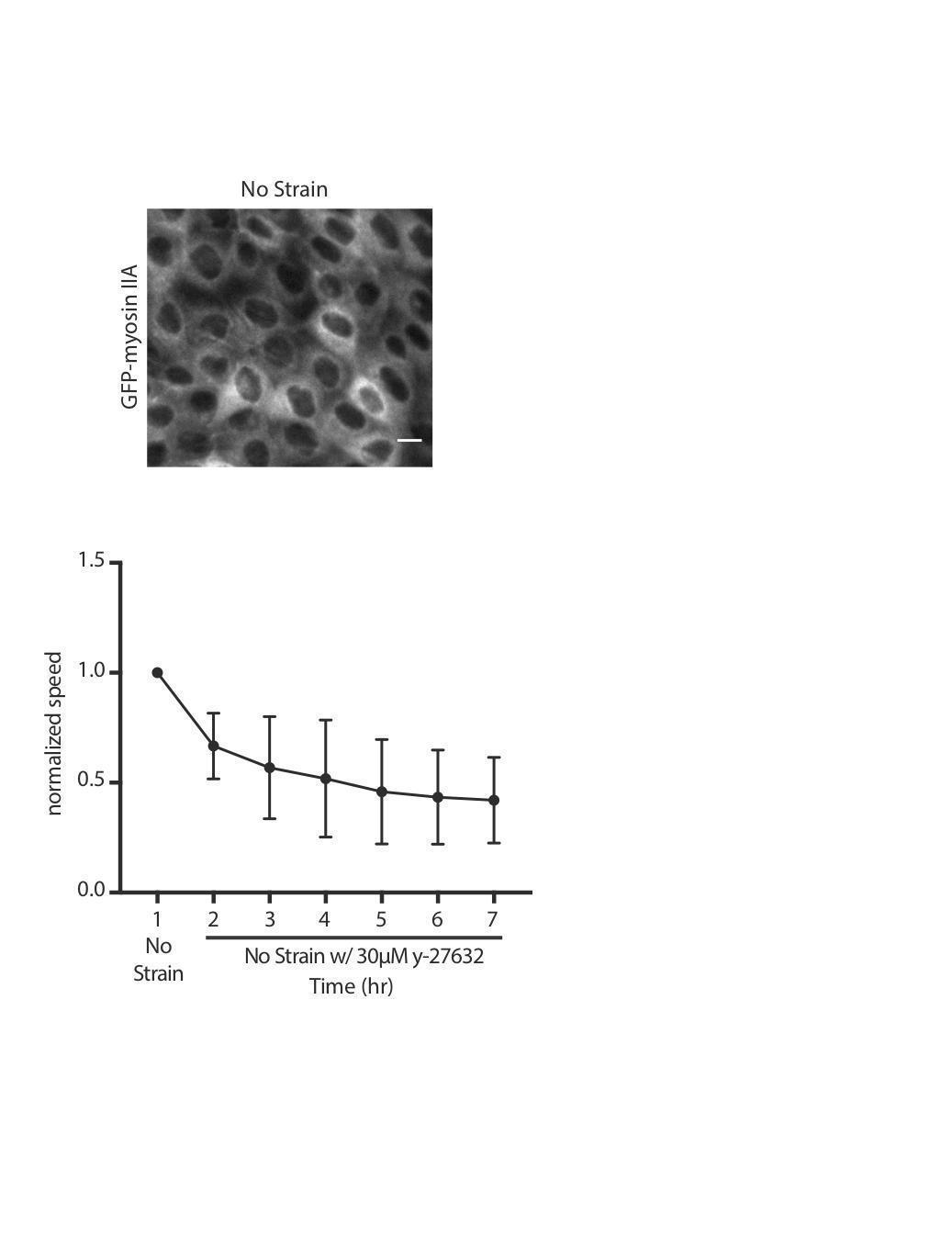


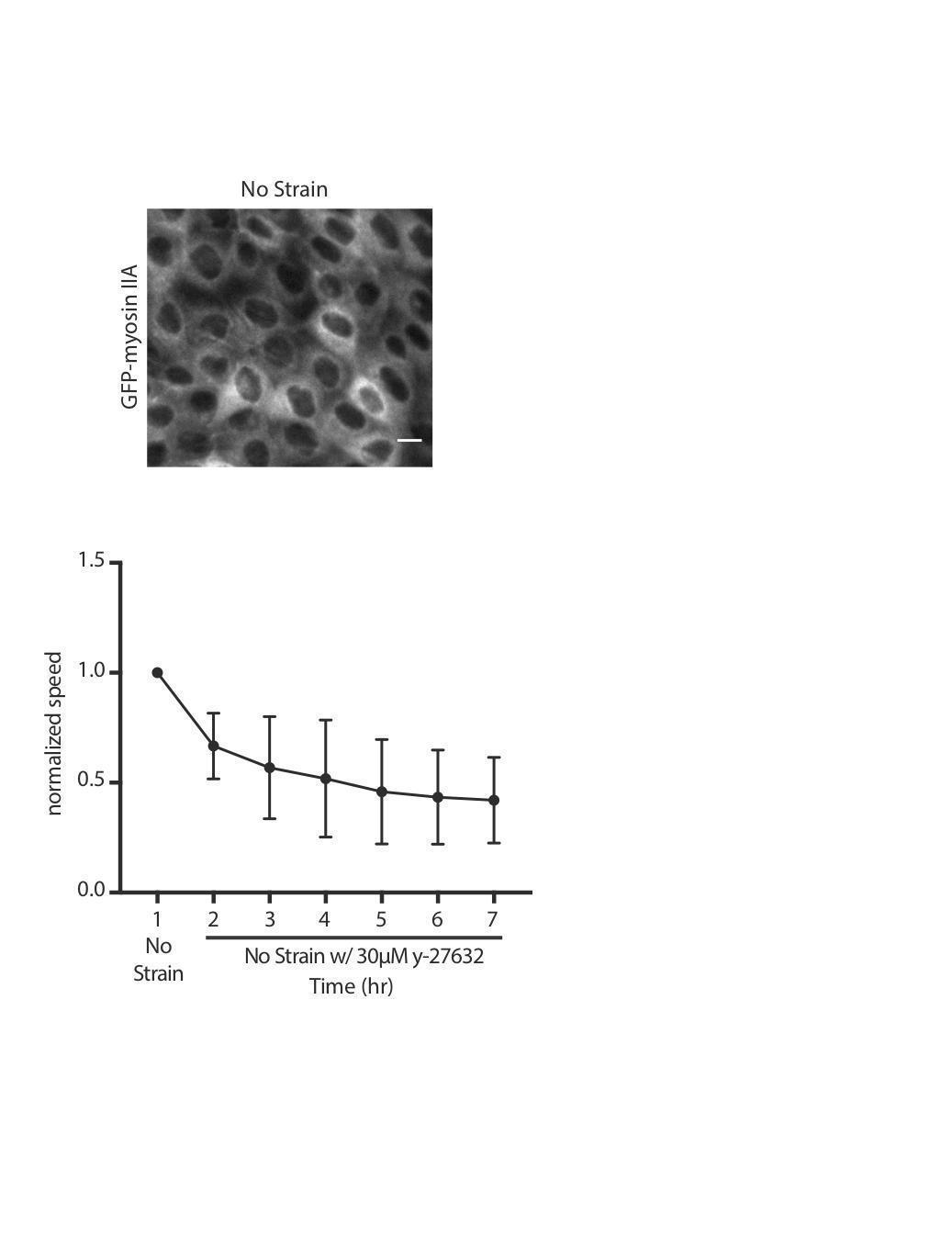


**Supplemental Figure 5:**

A. Representative image of MDCK cells expressing GFP-Myosin IIA under no strain where we do not see the accumulation of GFP myosin at the cortex of cells. Scale bars: 10μm

B. Normalized mean velocities over the course of 7 hr of imaging every 10 minutes with no strain and the ROCK inhibitor (30 μM y-27632). Hours 2–7 were normalized to the first hour of no strain and no inhibitor for all experiments. Quantifications were mean +/- SD from 3 independent experiments.

**
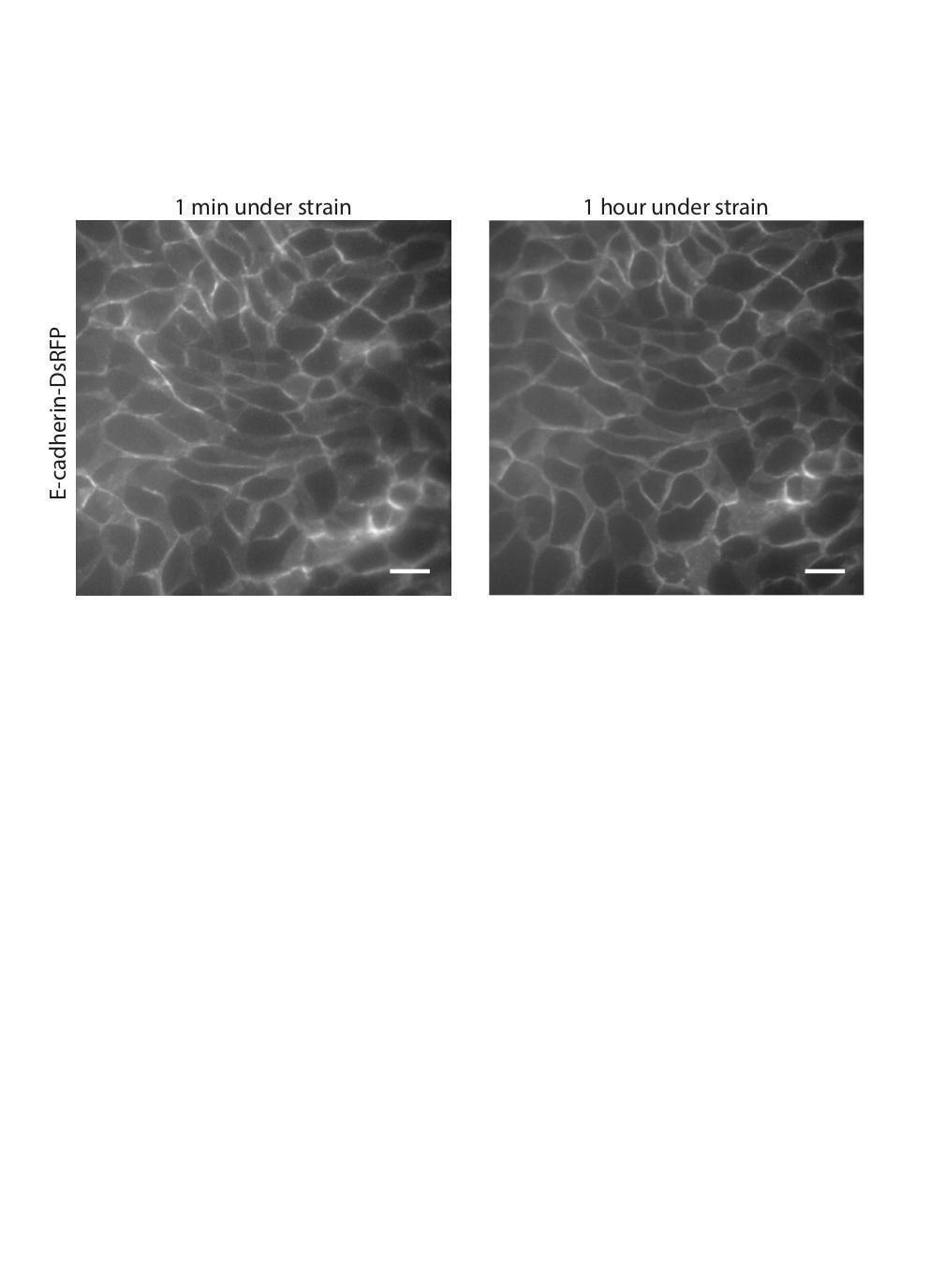
**

**Supplemental Figure 6:**

Representative images of MDCK cells expressing E-cadherin-DsRFP under 1 min and 1 hr of strain where we see no changes in E-cadherin. Scale bars: 10μm.


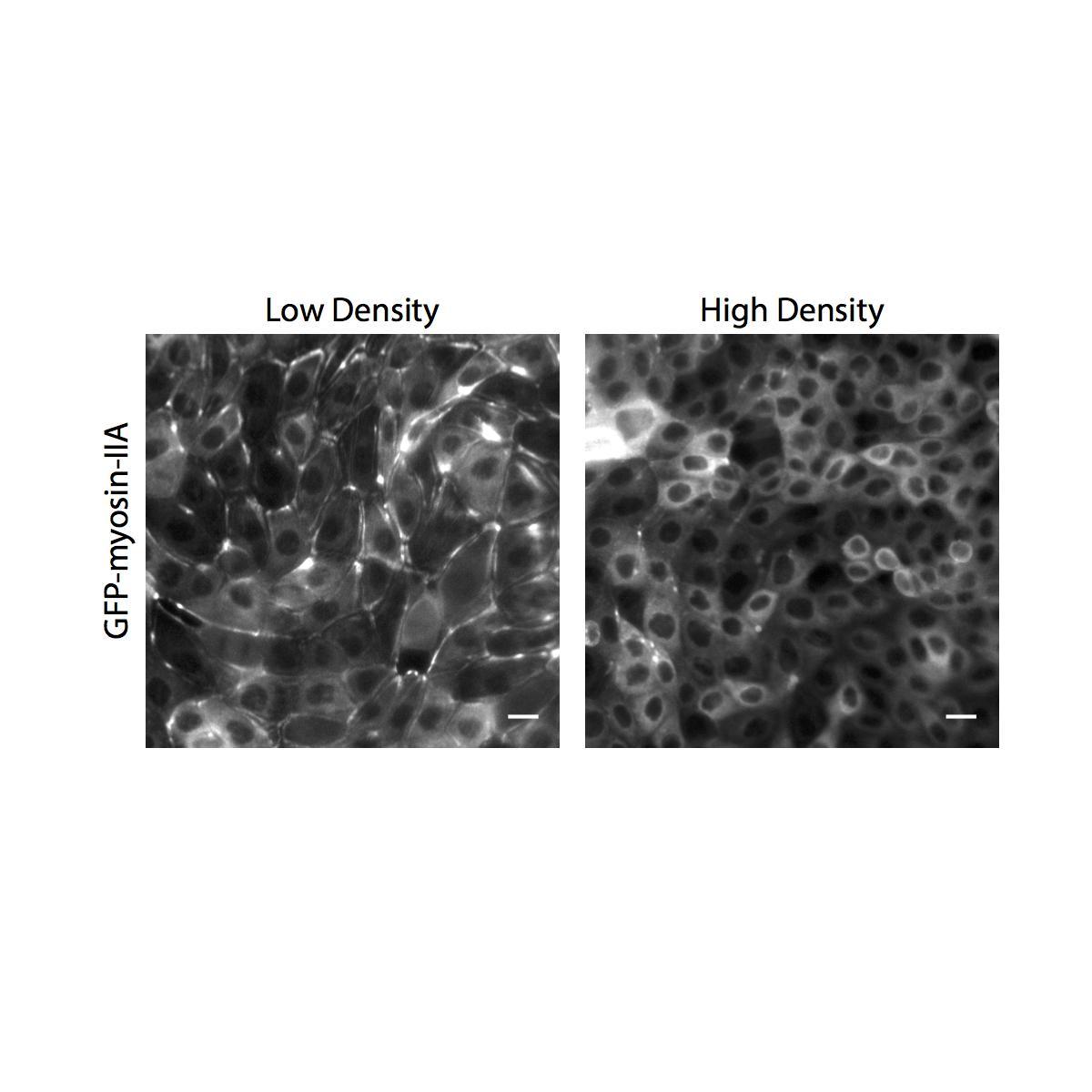


**Supplemental Figure 7:**

Representative image of MDCK cells expressing GFP-Myosin IIA grown at low and high density under 15% strain for 1 hr. Scale bars: 10μm.


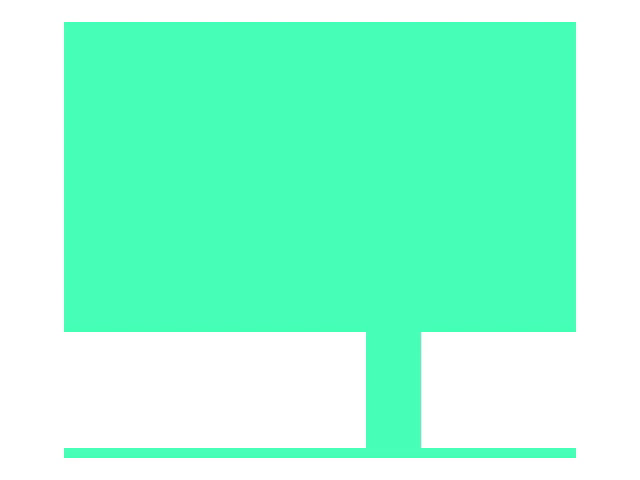


**Supplemental Movie 1:**

2D FEA simulation of the strain applied to the cell culture membrane from 0 kPa to 70 kPa of vacuum pressure to the side chambers using COMSOL Multiphysics (version 4.4, COMSOL Inc.).

**
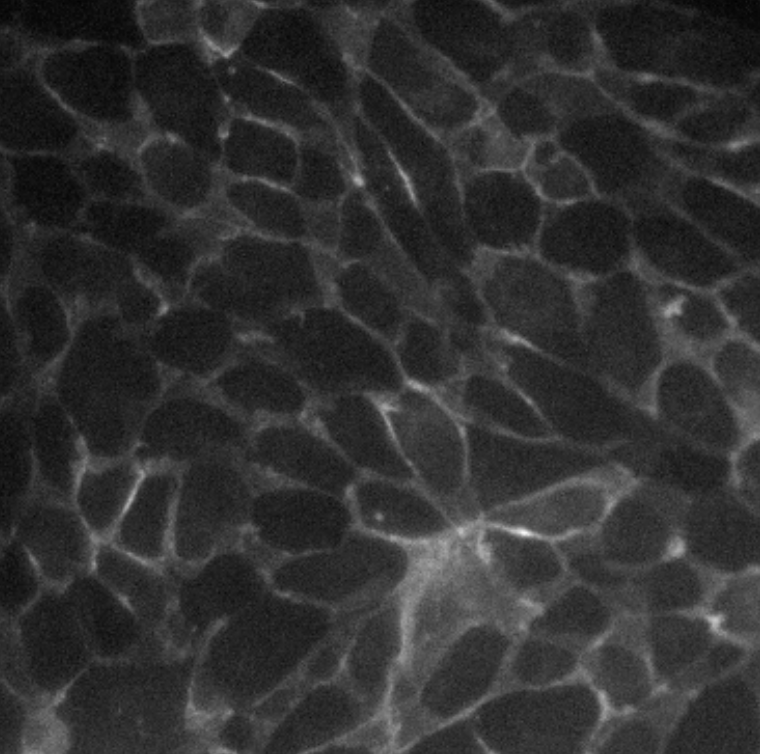
**

**Supplemental Movie 2:**

E-cadherin-RFP MDCK cells plated 20 hr before imaging on the cell stretching device. Image acquired every 3.5 kPa from 0 kPa to 70 kPa at 40x magnification at 5 sec interval between images.
